# Physicians’ perspectives on artificial intelligence in electrocardiography in clinical practice: a qualitative study

**DOI:** 10.1186/s12910-026-01572-7

**Published:** 2026-08-26

**Authors:** Gabriel Allgårdh, Gert Helgesson, Ulrik Kihlbom

**Affiliations:** Stockholm Centre for Healthcare Ethics (CHE), Department of Learning, Informatics, Management and Ethics, Karolinska Institutet, Stockholm, SE-171 77 Sweden

**Keywords:** Artificial intelligence, Electrocardiography, AI-ECG, Qualitative research, Medical ethics, Clinical decision-making

## Abstract

**Background:**

Artificial intelligence-enhanced electrocardiography (AI-ECG) may support ECG interpretation, prioritization, and workflow efficiency. ECGs are widely used, inexpensive, and often obtained with a low clinical threshold, meaning that AI-ECG may affect large numbers of patients and reshape an established clinical workflow. This study explored physician’s perspectives on integrating AI-ECG into clinical practice.

**Methods:**

Semi-structured interviews were conducted with 12 physicians in Region Stockholm, Sweden. Participants were selected through purposive and snowball sampling and included both specialists and nonspecialists. The interviews were transcribed and analyzed using thematic analysis.

**Results:**

Two main themes were identified: *Designing a new clinical landscape* and *Navigating a new clinical landscape*. Participants saw potential for AI-ECG to improve diagnostic accuracy, support prioritization, and reduce workload. At the same time, they raised concerns about overreliance, accountability, and the limitations of human oversight. Participants also emphasized that AI-ECG may generate incidental or prognostic findings beyond the original reason for obtaining an ECG, raising questions about disclosure, consent, and actionability.

**Conclusions:**

Informants viewed AI-ECG as a potentially useful support for ECG interpretation in high-volume clinical workflow, but also identified ethical, practical, and professional aspects that require consideration beyond technical performance alone. Because ECGs are common investigations and are often obtained with a low clinical threshold, AI-ECG should be evaluated with regard to its role as a core component of clinical workflow. Implementation should include real-world validation, attention to alert burden and deskilling, and protocols for incidental or prognostic findings.

**Supplementary Information:**

The online version contains supplementary material available at 10.1186/s12910-026-01572-7.

## Background

In recent years, artificial intelligence (AI) has attracted increasing attention in healthcare [1] and has increasingly been developed and implemented in the clinical setting. AI may improve diagnostic accuracy, workflow efficiency, and personalization of care [2]. However, its integration into clinical practice raises professional, societal, and ethical questions [3]. These questions are particularly relevant when AI systems are introduced into routine clinical workflows where clinicians remain responsible for interpretation and action.

Recent advances in machine learning have enabled automated analysis of complex clinical data [4]. Deep learning, a subset of machine learning, uses multilayered artificial neural networks to identify patterns that may not be captured by traditional rule-based methods [5]. These techniques have been applied across several areas of medicine, including medical imaging and cardiology [6–10]. In electrocardiography, this development builds on a longer history of computer interpreted ECGs (CIEs), which have supported clinical ECG interpretation through more conventional automated methods [11, 12].

The distinction between CIE and AI-enhanced ECG (AI-ECG) is important. Current CIE systems are already familiar to many clinicians and provide useful context for understanding how automated ECG interpretation is perceived in practice. However, AI-ECG systems may extend beyond conventional ECG interpretation by using large datasets and machine-learning methods to identify patterns associated with present or future disease [13, 14]. Physicians’ views on AI-ECG may therefore reflect both direct experience with existing CIE systems and anticipatory judgements about a more advanced, hypothetical, diagnostic tool.

Integrating AI solutions into electrocardiography has shown considerable promise [15, 16]. The ECG is a widely available, cost-effective, and noninvasive diagnostic tool [17]. It is used in emergency care, primary care, and inpatient settings, often with a low threshold. At the same time, accurate ECG interpretation requires clinical training and experience and remains vulnerable to human error [18]. AI-ECG systems could support interval and rhythm assessment, detection of acute abnormalities, prioritization of ECGs requiring urgent review, and comparison with previous recordings. They may also identify subtle patterns or predict conditions not evident to the human interpreter [19–25]. In selected ECG-based tasks, AI models have matched or exceeded medical-reader performance in controlled evaluations, and emerging pragmatic studies suggest clinical benefit in some workflows. By analyzing large datasets holistically without requiring mechanistic knowledge of pathophysiology, AI-ECG also opens new opportunities for preventive interventions [17, 26]. However, real-world effectiveness remains task- and implementation dependent [27, 28].

The development of AI-ECG raises ethical and operational challenges. Many machine-learning systems are difficult to interpret and create a so-called black-box problem that may limit clinicians’ ability to understand, justify, or contest AI-generated recommendations [29–31]. If neither clinicians nor developers can adequately explain an algorithm’s reasoning or determine whether its output was erroneous, questions of responsibility and legal liability are likely to arise in cases of patient harm [32]. Bias in training data may also lead to misdiagnoses or suboptimal care, particularly among underrepresented populations [33, 34], which in turn could perpetuate, or worsen, existing health disparities. Over time, the reliance of AI may also contribute to the deskilling of clinicians by reducing the number of opportunities for independent diagnostic reasoning [35].

Several strategies have been proposed to address these concerns. *Human-in-the-loop* models aim to preserve clinical responsibility and trust by ensuring that a clinician reviews and validates AI outputs [36–38]. However, human oversight does not by itself eliminate the risk of inappropriate reliance on automated recommendations. Clinicians may be vulnerable to automation bias, in which users over-rely on automated outputs even when these are incorrect or require critical verification. This risk may persist even in explainable systems, since explanations can increase perceived acceptability without necessarily improving decision accuracy and may, depending on their design, reinforce misplaced trust [39]. Explainable AI may therefore be useful, but current approaches remain limited and may provide misleading reassurance. These concerns suggest that human oversight and explainability should be accompanied by interface and workflow designs that promote active verification, critical engagement, and calibrated reliance rather than passive acceptance of AI output [40, 41]. Hybrid models may also help combine the flexibility of large language models with the transparency, reproducibility, and auditability of rule-based systems [42].

From a regulatory perspective, the European Union’s Artificial Intelligence Act [43] represents an attempt to govern AI in high-risk sectors such as healthcare. By emphasizing human-centered [44] and trustworthy [45] AI, and prioritizing fundamental rights and patient safety, the Act highlights the importance of ethical considerations in AI deployment. AI-ECG systems that constitute medical device software requiring third-party conformity assessment fall within the high-risk category under Article 6(1) of the AI Act. Healthcare providers implementing such systems must therefore ensure appropriate use, staff training, human oversight, performance monitoring, data quality, and incident reporting in accordance with the Medical Device Regulation (EU) 2017/745.

Evidence on physicians’ perspectives regarding AI-ECG remains limited. Since clinicians will be the end-users of such systems [46], their views are important for understanding how AI-ECG might be implemented safely, ethically, and practically. The aim of the present study was therefore to explore physicians’ perspectives on the integration of AI-ECG into clinical practice.

## Methods

### Study design and participants

We conducted a qualitative study using semi-structured interviews, a method chosen to explore physicians’ perspectives in depth while allowing participants to elaborate on relevant topics [47–49]. The Consolidated Criteria for Reporting Qualitative Research (COREQ) [50] was followed to ensure standardized reporting. Eligible participants were licensed physicians employed in Region Stockholm who interpreted electrocardiograms daily or near daily.

A sample size of at least 10 participants was sought to capture diverse perspectives and achieve information power. Recruitment combined purposive and snowball sampling [51–54]. Four participants were recruited directly through email invitations, and eight additional participants were identified through referrals from initial respondents. In total, 12 physicians were included, none of whom dropped out after consenting. One physician turned down the interview request.

### Data collection and interview guide

The interview guide was developed iteratively. Initial topics were identified from previous studies in related fields. The guide was then piloted with medical students, and their feedback was used to clarify wording and ensure that the questions aligned with the study objectives. The full interview guide is available as Supplementary file 1.

Table 1 summarizes the sample characteristics. The sample included 12 physicians employed in Region Stockholm with a balanced distribution by gender and specialist certification status. Specialists and nonspecialists from several clinical fields, including cardiology, general surgery, family medicine (of whom all were specialists), internal medicine, pediatrics, and emergency medicine. In this paper, a *specialist* refers to a physician who had completed formal specialist training (typically 5–7 years of residency) and obtained specialist certification in their field. A *nonspecialist* refers to a physician who had completed their medical degree but had not yet obtained specialist certification, including residents and other junior physicians, irrespective of clinical field. It should be noted that, in Sweden, a *general practitioner* is a physician with specialist certification in family medicine. All participants interpreted ECGs daily or near-daily, and their median ECG interpretation experience was 10 years, with a range of 2–40 years. All participants routinely encountered current CIE systems in their clinical ECG work. The participants used the same CIE system within Region Stockholm, meaning that their accounts of current CIE referred to the same type of automated ECG interpretation. One participant stated that he did not actively use CIE when interpreting ECGs. However, the CIE interpretation was still visible to him in the clinical workflow. Thus, non-use referred to not relying on the interpretation rather than to lack of exposure or experience. None of the participants had used AI-ECG systems in clinical practice. Between February 19 and April 22, 2024, the first author (GA) conducted semi-structured interviews with the study participants. Interviews were conducted in Swedish via Microsoft Teams and audio-recorded using a handheld device. Only the interviewer and the respective participant were present. No repeat interviews were conducted. The emergence of new categories leveled off after 11–12 interviews, and data saturation was therefore considered likely to have been reached. Recordings were transcribed verbatim manually by the first author in Microsoft Word, with Microsoft Excel used to organize excerpts. Quotations selected for publication were translated from Swedish into English by the first author. To support accuracy and idiomatic phrasing, an additional AI-assisted translation was generated using ChatGPT-4. This translation was compared with the author’s translation, and the final wording was revised where AI-generated suggestions improved faithfulness to the original meaning.

**Table 1:** Sample characteristics

Sample	Informants
Gender	Women (*n* = 6) Men (*n* = 6)
Duration	Average duration was approximately 31 min (27–57 min)
Specialist certification status	Physicians with specialist certification (*n* = 6) Physicians without specialist certification (*n* = 6)
Clinical role/field	Cardiology (*n* = 4), general surgery (*n* = 3), family medicine (*n* = 2), internal medicine (*n* = 1), pediatrics (*n* = 1), emergency medicine (*n* = 1)
ECG interpretation frequency	Daily (*n* = 9) Near-daily (*n* = 3)
ECG interpretation experience	Median experience was approximately 10 years (2–40 years)

### Data analysis

We analyzed the data using thematic analysis, following Braun and Clarke’s approach [47]. The aim was to identify explicit meanings expressed by participants through a bottom-up, inductive process.

First, three transcripts considered representative of the dataset were independently coded by the authors (GA, UK, GH). These initial analyses were compared and synthesized to create a preliminary coding framework, which was then applied to the remaining transcripts. During the familiarization phase, transcripts were read and reread, with notes and potential meaning units documented. Approximately 170 meaning units were identified and condensed. Similar units were grouped into subcategories, which were further abstracted into categories and themes through an iterative process of condensation and recategorization. Themes, categories, and subcategories were defined to maximize external heterogeneity (clear distinctions between groups) and internal homogeneity (coherence within groups) [47].

### Ethical considerations

The study posed no risk of physical harm to participants but did involve potential risks to personal integrity through breaches of confidentiality. To minimize these risks, all audio files were stored locally on a recording device and were deleted after transcription. Transcripts were anonymized by removing names, pronouns, and workplace identifiers, and each transcript was assigned a random numerical code. Anonymized transcripts were stored on a server requiring multifactor authentication. The study was conducted in accordance with the Declaration of Helsinki. Ethical approval was obtained from the Swedish Ethical Review Authority (No. 2023-07365-01). All participants gave informed consent to be interviewed and audio-recorded.

## Results

The analysis resulted in two overarching themes, six categories, and 19 subcategories (Table 2). The themes, *Designing a new clinical landscape* and *Navigating a new clinical landscape*, are described below with illustrative findings.

**Table 2:** Overview of themes, categories, and subcategories

Themes	Categories	Subcategories
Designing a new clinical landscape	Views of current CIE systems	*Current CIE is powerful in specific areas*
		*Skepticism towards current CIE performance*
		*Performance variability undermines CIE trustworthiness*
	Potential and risk of adopting AI-ECG	*Potential for AI-ECG*
		*Planning and estimating AI-ECG interpretation*
		*AI-ECG interpretation occurs in a clinical context*
		*Risk for overreliance on AI-ECG*
Navigating a new clinical landscape	Building trust and security	*Understanding interpretations made by AI-ECG*
		*Algorithmic bias*
		*Data protection*,* cybersecurity*,* and accessibility*
		*Building trust through validation and evaluation*
		*Combating AI’s lacking transparency*
	Responsibility and accountability	*A human-in-the-loop*
		*Ethical accountability and legal liability*
	Patient-practitioner relations	*The physician as mediator between the patient and AI-ECG*
		*Patient reception of AI-ECG*
		*Uniqueness of the human physician*
	Anticipated impact on physicians’ roles and professional identity	*Managing Prognostic and Incidental Findings with AI-ECG*
		*Adapting to a Rapidly Evolving Clinical Landscape*

### Theme 1: designing a new clinical landscape

This theme reflects physicians’ perspectives on current computer-interpreted electrocardiography (CIE) and the potential integration of AI-ECG in clinical practice.

#### Views of current CIE

Participants reported both strengths and limitations of existing CIE systems. Several noted their reliability in classifying normal ECGs and measuring specific intervals such as PQ and QRS durations [Informants 6, 3]. Others appreciated the system’s high sensitivity for severe abnormalities.

Concerns included excessive sensitivity leading to false alarms, lack of specificity, and potential overreliance by inexperienced clinicians. One participant expressed complete distrust and chose not to use CIE at all [Informant 2]. Several emphasized the importance of knowing the systems’ strengths and weaknesses to integrate them responsibly into practice.

Quotes and subcategories of the *Views of current CIE* category are provided in Table 3.

**Table 3:** Data extracts for category: views of current CIE systems

Subcategory	Data extracts
Current CIE is powerful in specific areas	*For example*,* when interpreting what is a normal ECG*,* one could*,* in principle*,* also use today’s algorithms. If it says it’s normal*,* it is likely normal.* *[6]*
Skepticism toward current CIE performance	*I don’t look at it. […] It says*,* ‘Nonspecific ST-T changes*,*’ and I think*,* ‘Those ST- T changes I have already cleared*,*’ and then I get irritated when I see the interpretation…* *[12]*
CIE’s output may be misleading	*Morphologically*,* it might resemble a particular diagnosis*,* but when you as a human consider the bigger picture*,* it’s more likely to be another diagnosis.* *[2]*

#### Potential and risk of adopting AI-ECG

Participants identified several potential benefits of AI-ECG, including enhanced objectivity, reduced diagnostic variation, and increased patient safety [Informant 5]. AI-ECG was seen as particularly valuable for supporting less experienced physicians and reducing administrative burdens [Informant 1]. Some participants framed the adoption of AI-ECG as an ethical obligation if it demonstrates superiority to current care, consistent with the principle of beneficence [Informant 11].

Nonetheless, participants stressed the importance of cautious, stepwise implementation to allow early errors to be detected and corrected [Informant 3]. Concerns included overreliance [Informant 10], resistance from experienced clinicians [Informant 6], and skepticism from governing bodies [Informant 11]. Some informants highlighted that AI-ECG should complement, rather than replace, broader clinical assessments that incorporate symptoms, biomarkers (e.g., troponin), and clinical judgment [Informant 12]. Human skills in empathy, holistic evaluation, and patient communication were considered irreplaceable by some [Informant 7].

Quotes and subcategories of the *Potential and risk of adopting AI-ECG* category are provided in Table 4.

**Table 4:** Data extracts for category: potential and risk of adopting AI-ECG

Subcategory	Data extracts
Potential for AI-ECG	*Interpreting what is a normal ECG […] if it says normal*,* it would probably be normal.* *[8]* *AI […] can recognize morphological patterns that are extremely difficult for humans to identify […] there are certainly many morphological diagnoses that will become much more precise.* *[2]*
Considerations on when to implement AI-ECG	*We have an ethical obligation to use AI-ECG if we have conclusively seen that it is better for patients.* *[5]* *After things have settled down and everyone seems to agree that the technology is sound*,* I would start looking into it. I would probably remain skeptical for quite a while […] and then likely join those who use it reasonably.* *[3]*
AI-ECG interpretation occurs in a clinical context	*You would need to connect the ECG interpretation with other parameters…* *[12]*
Risk for overreliance on AI-ECG	*Things can go wrong if we let go of human judgment*,* the human eye*,* and our instinct for assessing a patient and their condition.* *[7]*

### Theme 2: navigating a new clinical landscape

This theme captures participants’ reflections on trust, responsibility, patient–practitioner interactions, and the evolving professional role in the context of AI-ECG.

#### Building trust and security

Transparency was regarded as essential for trust in AI-ECG [Informant 5]. Opaque algorithms were compared to “oracles”, raising concerns about undermining doctor–patient communication [Informant 11]. Some participants favored explainable AI as a means of improving transparency [Informant 9]. In some cases, participants stated that human evaluation is imperative when assessing certain conditions, such as atrial fibrillation [Informant 1, 12]. Others noted that complete transparency may be neither feasible nor necessary, drawing parallels to existing laboratory tests, such as troponin [Informant 5].

Concerns also included algorithmic bias [Informant 10], cybersecurity threats [Informant 6], and data-sharing ethics [Informant 12]. Suggestions included oversampling underrepresented groups during training [Informant 9], implementing strong data protection [Informant 1], and avoiding undue commercial influence [Informant 3]. Some participants emphasized the need for rigorous validation [Informant 10] and ongoing surveillance to ensure reliability across diverse populations [Informant 5, 6].

Quotes and subcategories of the *Building trust and security* category are provided in Table 5.

**Table 5:** Data extracts for category: building trust and security

Subcategory	Data extracts
Understanding interpretations made by AI-ECG	*In healthcare*,* where stakes are high*,* we need to understand the basis of the algorithm’s decisions—specifically*,* which part of the ECG it has considered.* *[5]*
Algorithmic bias	*There could be many external factors that the computer isn’t aware of*,* which can make it challenging to consider*,* and therefore*,* the assessments might be unreliable.* *[10]*
Data protection, cybersecurity, and accessibility	*I’d rather have a well-trained*,* high-performance AI. At the same time*,* […] we document sensitive things in medical records*,* […] but you wouldn’t want to omit data points due to cybersecurity and thereby decrease the quality of the AI* *[1]*
Validation and evaluation are conditions for trust	*Bias needs to be evaluated. We need to have trust in the systems*,* but there also needs to be an evidence-based approach. This applies to all technology whether it’s AI or not.* *[6]*
Combating AI’s lacking transparency	*I want more solid evidence than relying on an AI that suggests atrial fibrillation. For other processes*,* maybe it’s enough to get an answer*,* like the CRP level.* *[12]* *[…] but then it becomes very important that it has been validated*,* and that it is clear in which situations one may rely on that interpretation instead of interpreting it oneself or consulting someone else.* *[6]*

#### Responsibility and accountability

Participants agreed that AI-ECG should support, not replace, physicians [Informant 1, 3]. Some emphasized that clinicians must retain ultimate responsibility for decisions [Informant 8]. Some likened AI-ECG outputs to laboratory results, requiring physician verification [Informant 2]. Also, “a human-in-the-loop” was proposed as a strategy to help combat faulty AI suggestions as well as a way to ascertain human accountability [Informant 8]. While AI-ECG could detect rare conditions [Informant 7], participants warned that increased output might also increase physicians’ workload by generating additional findings that require assessment [Informant 1].

Quotes and subcategories of the *Responsibility and accountability* category are provided in Table 6.

**Table 6:** Data extracts for category: responsibility and accountability

Subcategory	Data extracts
The need of a human-in-the-loop	*Certain parameters have been entered into the machine-learning algorithm*,* but you do not know how it arrived at that particular conclusion. In that case*,* it is you*,* as the physician*,* who should be making the treatment decision—not the AI. For now*,* AI should be seen as a tool rather than taking over responsibility.* *[5]* *It could provide helpful insights in identifying issues that were initially overlooked. AI makes suggestions and puts you on the spot—then you must consider for yourself: “Is the computer correct or not?”* *[2]*
Ethical accountability and legal liability	*In some ways*,* the algorithm’s end-user becomes responsible. The developers have the accountability to ensure it functions properly. Still*,* if the patient and I reach an agreement*,* then I can accept the accountability for it.* *[8]* *An AI output must function like a laboratory result. It has to appear in your verification inbox*,* and it should not be considered valid until a physician has assessed the result in relation to the rest of the clinical information and signed it. In that case*,* the physician who verifies the result is also the one responsible if the diagnosis turns out to be incorrect.* *[2]*

#### Patient–practitioner relations

Participants anticipated both opportunities and challenges in doctor–patient relationships. While some expected patients to respond positively to AI-ECG [Informant 8], others predicted skepticism and confusion if results were not clearly explained [Informant 2, 3]. Physicians highlighted their role in interpreting and communicating AI findings, emphasizing transparency and patient education [Informants 6, 8, 11]. Misaligned expectations could lead patients to seek multiple opinions, complicating continuity of care [Informant 1].

Several informants noted that empathy, shared decision-making, and nuanced communication remain beyond AI’s scope [Informants 2, 5, 11].

Quotes and subcategories of the *Patient–practitioner relations* category are provided in Table 7.

**Table 7:** Data extracts for category: patient–practitioner relations

Subcategory	Data extracts
The physician as mediator between the patient and AI-ECG	*I don’t foresee a scenario where AI interpretation directly suggests treatments and where I find myself disputing with a patient whether the AI is correct or not.* *[3]* *There may be specialist interest associations that can provide ethical guidelines in advance. If a patient has an agenda*,* one can rely on these guidelines: “This is what the collegium states”. I don’t wish to sit entirely alone as a doctor. There should be guidance—reports*,* on what should be done.* *[8]*
Patient attitudes to AI-ECG	*I sense that it would not diminish trust in healthcare.* *[8]* *I believe there will be a faction that remains skeptical of computers. I also think there will be a group that absolutely wants to be assessed by AI*,* and then there will be some in between*,* with a normal distribution around the median.* *[2]*
Uniqueness of the human physician	*The ability to perceive the whole person*,* how body language*,* voice level*,* and other non-verbal cues contribute to a comprehensive assessment of the patient’s health condition. […] There are subtle nuances in a human communication that are difficult for AI to capture.* *[5]* *The human touch*,* making a person feel warm*,* cared for*,* and secure*,* and examining a patient in an empathetic and dignified manner*,* are aspects AI cannot currently replicate.* *[5]*

#### Anticipated impact on physicians’ roles and professional identity

Participants reflected on how AI-ECG might reshape physicians’ roles. To some participants, potential benefits included focusing on *human-centered* aspects of care [Informant 3]. Concerns included deskilling and increased dependence on technology, underscoring the need to maintain clinical expertise and focus on patient outcomes rather than diagnostic processes [Informant 7]. Also, career paths might be less straightforward with AI. This could potentially lead to occupational stress for junior doctors [Informant 2].

Ethical dilemmas related to incidental findings were also raised. Some emphasized a duty to disclose actionable findings, drawing parallels to genetic testing for BRCA1/2 mutations [Informant 2], while cautioning against undue stress from uncertain results [Informant 1]. Clear protocols for disclosure were considered essential [Informant 9].

Quotes and subcategories of the *Anticipated impact on physicians’ roles and professional identity* category are provided in Table 8.

**Table 8:** Data extracts for category: Anticipated impact on physicians’’ professional role and identity

Subcategory	Data extracts
Managing prognostic and incidental findings with AI-ECG	*Comparing the use of AI-ECG with the case of individuals carrying the BRCA1/2 mutations*,* we see parallels in decision-making under uncertainty.* *[2]* *Let’s consider a patient who is found to have an increased risk of developing Huntington’s disease based on an ECG taken at the age of 22. How is this patient supposed to react? How should they live their life?* *[1]* *…if it is possible to prevent or delay the condition*,* then it is unethical not to inform the patient.* *[2]*
Adapting to a rapidly evolving clinical landscape	*We cannot assume that the role of a surgeon today will be the same in five years. AI could very well make an entrance in a way we cannot currently predict. […] The medical profession is built on investing many years into highly specialized fields—it’s not simply a matter of switching roles. […] A specialty that suits a certain type of person today may not suit them tomorrow.* *[2]* *I view AI’s role broadly as a facilitator that allows doctors to engage in even more rewarding medical activities. Physicians can then devote more time to other aspects*,* such as communicating with patients*,* providing detailed information in a calm setting*,* enhancing human interaction*,* pursuing further education*,* and teaching.* *[3]*

## Discussion

This study explored physicians’ views on the use of AI-enhanced electrocardiograms in clinical practice. The main contribution of the study is how the general concerns about AI could take specific form in the ECG setting. Participants described AI-ECG as a technology that would enter an already high-volume, low-threshold, and partly automated clinical workflow. Their reflections therefore point toward implementation questions that are specific to the ECG setting: what kinds of ECG tasks should be automated, how AI-generated findings should be prioritized, when physicians should be expected to verify the output, and how incidental or prognostic information should be handled.

Although many of the ethical issues identified in this study—opacity, overreliance, deskilling, accountability, and biased training data—are familiar from the wider literature on AI in healthcare, the ECG setting lends itself to a nuanced discussion of these ethical questions. The ECG context is ethically distinctive because ECGs are inexpensive, rapidly acquired, widely available, and deeply integrated into routine and emergency care. In many acute settings, ECGs are obtained not only when a cardiac diagnosis is strongly suspected, but also as part of a broad initial assessment. AI-ECG could therefore affect many patients, including patients who did not actively seek evaluation for a cardiac or prognostic condition. The ethical implications are shaped not only by algorithmic performance, but also by the frequency and low threshold of ECG use.

AI-ECG is also distinctive because ECGs are not only widely used but also repeated over time. These features make the ECG a particularly suitable context for automated measurement, longitudinal comparison, and large-scale deployment. In practice, AI-ECG could support or partly take over tasks currently performed by clinicians or conventional CIE systems, including measurement of intervals and axes, rhythm and conduction classification, detection of acute abnormalities, prioritization of ECGs requiring urgent review, comparison with previous recordings, and prediction of conditions not evident to the human interpreter. However, if AI-ECG expands the ECG from a diagnostic test into a broader predictive tool, it may also change the role of the ECG in clinical practice. A recording obtained for one clinical reason may generate information about future risk, latent disease, or unrelated conditions, thereby blurring the boundary between clinical diagnostic testing, opportunistic screening, and prognostic risk assessment.

### Human interaction and the patient–physician relationship

Earlier research has suggested that digitalization in healthcare may erode important social interactions. For example, a 2018 study showed that electronic medical records disrupted professional rituals and threatened the patient–physician relationship [55]. The present study adds a more specific observation from the ECG setting. Several informants did not view AI-ECG primarily as a threat to patient contact. Instead, they suggested that if AI-ECG could reduce time spent on routine technical interpretation, sorting, or documentation, physicians might have more time for direct patient contact.

This point is relevant because ECG interpretation is often a high-volume activity. Clinicians may spend substantial time reviewing normal, low-risk, or technically routine ECGs, particularly in acute and inpatient settings. If AI-ECG could safely help sort, prioritize, or pre-interpret such ECGs, it might reduce cognitive and administrative workload. However, the interviews also suggest a condition for this benefit: the system must reduce work rather than create additional tasks. If an AI-ECG generates outputs that physicians must repeatedly verify, interpret, or document despite uncertain immediate clinical value, they may increase rather than reduce workload.

Concerns about empathy and human connection nevertheless remain. A 2020 study concluded that AI will not develop genuine empathy in the foreseeable future [56]. This was consistent with participants’ views. They did not see technical performance as a substitute for the relational and interpretive aspects of medical care. This becomes important if AI-ECG produces unexpected or prognostic information, since communicating uncertain risk requires clinical judgement, interpersonal skill, and understanding of the clinical context.

### Safety, reliability, and the need for validation

The role of AI in healthcare has been described as augmentative, combining human expertise with machine intelligence [38]. In the interviews, informants saw possible safety benefits from AI-ECG, especially if it could detect subtle abnormalities, prioritize urgent ECGs, or support clinicians with less cardiology experience. At the same time, they were cautious about whether these benefits would appear in everyday practice.

ECGs are frequently used in clinical encounters, especially in emergency care. AI-ECG could therefore influence triage, diagnostic pathways, and the urgency assigned to a patient. A false negative could delay treatment of time-sensitive disease, while a false positive could lead to unnecessary alarm, investigations, monitoring, or admission. The interviews suggest that physicians are not only asking whether AI-ECG is accurate in controlled studies, but whether it can be trusted in the clinical situations where ECGs are actually used.

These findings suggest that validation of AI-ECG should reflect real clinical use [46]; overall accuracy is not enough. Evaluation should include emergency setting, low-prevalence conditions, clinically important subgroups, different ECG devices, variable signal quality, electrode misplacement, comorbidities, medication effects, and local workflows [57]. It should also assess downstream effects, such as alert burden, referral patterns, additional investigations, missed diagnoses, and clinician workload [58, 59].

Future physicians’ views of AI-ECG may be shaped by their experience of current CIE systems. Several informants described cases where current CIE systems either missed important abnormalities or produced false positives. This is important because physicians may not encounter AI-ECG as an entirely new technology, but as an extension of an already familiar and imperfect form of automation. Trust in future AI-ECG may therefore be influenced by earlier experiences with conventional CIE.

Some participants raised concerns about overreliance and deskilling. ECG interpretation is a core skill across many medical fields, and if clinicians increasingly defer to AI-ECG, the ability to independently recognize ECG patterns may weaken over time. A 2019 study found that primary care physicians misclassified cardiac events in nearly half of cases [18], underscoring the risk of relying on automated tools without sufficient human expertise.

### Transparency, ethics, and regulation

A recurring theme in our interviews was the opacity of algorithmic reasoning. Informants stated that if they do not understand how AI reaches its conclusions, they may be reluctant to trust or use the system. Participants did not necessarily demand full technical transparency in the sense of understanding every model parameter. Rather, they raise a more clinically practical question: what kind of explanation is needed for a physician to act responsibly on an AI-ECG output? This distinction matters. Clinicians already use many tests that they cannot fully explain at a technical level, including laboratory analyses and imaging techniques. The ethical issue may therefore not be opacity alone, but whether the output is sufficiently validated, clinically meaningful, communicated with uncertainty, and linked to a clear responsibility structure [60–63]. These concerns are reflected in policy frameworks such as the European Commission’s assessment list for trustworthy AI [45] and the European Union’s AI Act [43], both of which stress transparency, accountability, and safety.

However, the ECG setting makes oversight difficult if AI-ECG produces large numbers of alerts, risk estimates, or secondary findings. Physicians may become formally responsible for information that is difficult to verify, explain, or act upon. The interviews therefore suggest that transparency should not be understood only as model interpretability. It should also include clear reporting of performance, uncertainty, intended use, limitations, and clinical pathways for acting on the output.

### Incidental and prognostic findings

A further finding from the interviews concerns incidental or prognostic information. Several participants reflected on the possibility that AI-ECG could identify clinically relevant but unexpected risks, while also producing uncertain or difficult-to-interpret information. This raises questions about the duty to disclose, the patient’s right not to know, informed consent, and the boundary between diagnosis and screening. A patient may undergo ECG testing because of chest pain, syncope, dyspnea, preoperative assessment, medication monitoring, or as a part of a broad emergency evaluation. If the same ECG is then used to infer future disease risk or detect conditions unrelated to the reason for testing, the encounter may shift from ordinary clinical assessment toward opportunistic screening. This shift may occur without the patient having understood or consented to that broader use.

One participant drew parallels to genetic testing for BRCA1/2 mutations, where probabilistic information may have major implications for future health, family members, and preventive interventions. The analogy is useful because it shows that prognostic information may be clinically and ethically important even when it does not imply certainty of future disease. However, AI-ECG differs from genetic testing in important ways. BRCA testing is usually performed in a context where risk prediction is the explicit purpose, and where counselling and consent can be structured around that purpose. By contrast, an ECG is often obtained as a routine or acute diagnostic test. If AI-ECG is used to infer broader risks from such recordings, a test ordered for one clinical purpose may become a form of opportunistic screening.

This creates the need to distinguish between actionable and non-actionable findings. If an AI-ECG system identifies a validated, clinically actionable risk for which effective follow-up or preventive intervention exists, there may be a stronger ethical reason to disclose it. Conversely, disclosing uncertain, poorly validated, or non-actionable risk estimates may cause anxiety, unnecessary investigations, and harm without clear benefit. The obligation to inform patients should therefore depend not only on the technical ability to generate predictions, but also on the validity, clinical significance, actionability, and possible consequences.

These findings suggest that implementation of AI-ECG should be accompanied by explicit protocols for incidental and prognostic findings. Such protocols should clarify which findings should be disclosed, who is responsible for disclosure, how uncertainty should be communicated, and what follow-up pathways should be available. They should also address whether patients should be informed in advance that AI-ECG may generate information beyond the immediate reason for testing, and whether patients should have any possibility to decline receiving certain categories of prognostic information.

### Health equity and bias

Another issue raised by participants concerns fairness. AI-ECG may reduce some forms of human variation by offering more standardized assessments, but if training datasets are incomplete or unrepresentative, AI-ECG may reproduce or worsen existing inequities.

ECG patterns may vary with age, sex, ancestry, body habitus, comorbidities, medication use, and technical factors. If AI-ECG systems are trained or validated on populations that do not reflect the patients in whom they are deployed, errors may be unevenly distributed. Because ECGs are used so widely, even small performance differences across groups could affect many patients. Bias in AI-ECG may appear not only as missed diagnoses, but also as unequal rates of false alarms, unnecessary follow-up, or reassurance.

Ensuring fairness will require diverse and representative training datasets, continuous updating, and validation across clinical settings, ECG devices, and patient subgroups [16, 34]. It will also require monitoring of downstream consequences, including referral patterns, investigation rates, treatment decisions, and patient anxiety.

### Implications for implementation

The interviews point toward several practical considerations for implementation. First, AI-ECG should be introduced with a clearly defined clinical role. Participants were more receptive to AI-ECG as a tool for prioritization, second reading, measurement support, and detection of easily missed abnormalities than as an independent decision-maker. Second, AI-ECG outputs should be designed to support clinical workflow. A system that produces excessive alerts or poorly actionable risk estimates may undermine safety by increasing review burden and alert fatigue. Third, implementation should include education that preserves ECG competence, especially among nonspecialists. Clinicians need enough independent interpretive skill to detect obvious errors, question unexpected outputs, and understand when expert review is needed.

Fourth, AI-ECG requires governance for secondary uses of ECG data. If systems are used not only for immediate ECG interpretation but also for prognostic prediction, patients and clinicians need clearer expectations about what information may be generated and how it should be handled.

Finally, evaluation should continue after deployment. Since ECGs are used in many settings and patient groups, local monitoring of performance, alert burden, false positives and negatives, and downstream clinical effects should be part of implementation.

### Methodological considerations

This study’s strengths lie in its qualitative design, which allowed physicians to describe how AI-ECG might affect every day clinical work. The inclusion of both specialists and nonspecialists from different workplaces was important because ECG interpretation is distributed across several clinical fields. Semi-structured interviews made it possible to identify not only general attitudes toward AI, but also more specific concerns about ECG workflow and current CIE systems.

Several limitations should be acknowledged. The interview guide was developed by the authors and therefore inevitably reflected their prior assumptions about which aspects of AI-ECG were the most relevant to explore. Although the semi-structured format allowed participants to elaborate on their responses and raise additional issues, the use of predefined and standardized questions may have shaped the scope and direction of the interviews. This may have homogenized responses and foregrounded topics already emphasized in the existing literature or regarded by the authors as particularly important. Consequently, unexpected or spontaneously generated perspectives may have been less likely to emerge.

The study was restricted to Region Stockholm, Sweden, which may limit transferability to other healthcare systems or cultural contexts. Although purposive recruitment was used to include physicians with different professional roles, 8 of the 12 participants were identified through snowball sampling from four directly recruited physicians. This may have introduced network homogeneity, as participants recruited through professional contacts may have shared similar institutional experiences, attitudes, or assumptions about AI-ECG [64].

Participants were asked about their medical background, clinical role, and experience with ECG interpretation. However, we did not assess participants’ broader experience with AI, AI literacy, or baseline attitudes toward AI using a structured instrument. This is a limitation, since previous familiarity with AI, general trust or distrust in automation, and personal expectations about AI in healthcare may have influenced how participants interpreted the interview questions and framed their responses.

The emergence of new categories appeared to level off during the final interviews, and data saturation was therefore judged to have been reached. Since saturation was assessed close to the stopping point in a sample of 12 participants, this judgment should be interpreted cautiously. Empirical work on qualitative saturation suggests that saturation in interview studies is often reached within a relatively narrow range of interviews, particularly when the research objective is focused [53]. However, saturation should not be treated as a fixed numerical threshold. Sample adequacy depends on several study characteristics, including the complexity of the phenomenon, the sampling strategy, the structure of the interview guide, participant homogeneity, and the type of saturation being assessed [53]. In the present study the use of snowball sampling may have increased sample homogeneity as participants recruited through professional networks may have shared similar experiences or assumptions. This could have contributed to an earlier impression of saturation. Saturation should therefore be understood as a pragmatic assessment within the scope of this study rather than as evidence that all relevant views on AI-ECG had been captured.

Online video interviews may have reduced opportunities to observe nonverbal communication [65]. As with most qualitative studies, the researchers’ own perspectives may have shaped the interpretation of the data, and reliance on self-report introduces potential recall or social desirability biases [66]. Finally, some interview questions may have been leading in that they explicitly introduced possible concerns about AI-ECG rather than eliciting them entirely organically, which may have influenced how participants framed their responses.

## Conclusions

This study suggests that physicians see potential for AI-ECG to support ECG interpretation, prioritization, and workflow efficiency, while also identifying ethical and practical concerns that should be addressed before implementation. Because ECGs are widely used, inexpensive, and often obtained with a low clinical threshold, AI-ECG should be evaluated not only as a technical tool but also as a change to a core component of clinical workflow. Implementation should include real-world validation, clear limits on intended use, attention to alert burden and deskilling, and protocols for incidental or prognostic findings. Transparent performance reporting, fairness across patient groups, appropriate human oversight, and continuing medical education will be important for calibrated use.

## Supplementary Information

Supplementary Material 1.

## Data Availability

De-identified excerpts are available from the corresponding author on reasonable request.

## Abbreviations

AI: Artificial intelligence
AI-ECG: AI-enhanced electrocardiogram
CIE: Computer-interpreted electrocardiogram
