# Supplementary material for: Physicians’ perspectives on artificial intelligence in electrocardiography in clinical practice: a qualitative study
Source: BMC Med Ethics. 2026 Aug 26;27:175. doi: 10.1186/s12910-026-01572-7 (PMC13520325; doi:10.1186/s12910-026-01572-7)
Supplement: Supplementary file 1 [file 12910_2026_1572_MOESM1_ESM.docx]

**ADDITIONAL FILE 1. INTERVIEW GUIDE USED IN SEMI-STRUCTURED INTERVIEWS**

**Interview guide**
1. Could you describe your medical background and current clinical role?

2. How would you describe your experience and proficiency in interpreting electrocardiograms (ECGs)?

3. From an ethical standpoint, what do you perceive as the main advantages and disadvantages of AI-based ECG interpretation?

4. How do you think the general public might respond if AI-ECG were implemented in routine clinical practice?

5. What are your views on the importance of transparency in AI-generated interpretations?

6. How do you think AI-ECG may affect patient autonomy, confidentiality, and the principle of equal treatment?

7. What are your thoughts regarding professional accountability and legal liability when AI systems are involved in ECG interpretation?

8. Do you foresee any risks of overreliance on AI or potential deskilling among clinicians with the introduction of AI-ECG

9. Is there anything else you would like to add or emphasize that we have not discussed?
